# New Pathogenic Concepts and Therapeutic Approaches to Oxidative Stress in Chronic Kidney Disease

**DOI:** 10.1155/2016/6043601

**Published:** 2016-06-27

**Authors:** José Pedraza-Chaverri, Laura G. Sánchez-Lozada, Horacio Osorio-Alonso, Edilia Tapia, Alexandra Scholze

**Affiliations:** ^1^Department of Biology, Faculty of Chemistry, UNAM, 04510 Mexico City, DF, Mexico; ^2^Laboratory of Renal Physiopathology, INC Ignacio Chávez, 14080 Mexico City, DF, Mexico; ^3^Department of Nephrology, INC Ignacio Chávez, 14080 Mexico City, DF, Mexico; ^4^Department of Nephrology, Odense University Hospital, 5000 Odense, Denmark; ^5^Institute of Clinical Research, University of Southern Denmark, 5000 Odense, Denmark

## Abstract

In chronic kidney disease inflammatory processes and stimulation of immune cells result in overproduction of free radicals. In combination with a reduced antioxidant capacity this causes oxidative stress. This review focuses on current pathogenic concepts of oxidative stress for the decline of kidney function and development of cardiovascular complications. We discuss the impact of mitochondrial alterations and dysfunction, a pathogenic role for hyperuricemia, and disturbances of vitamin D metabolism and signal transduction. Recent antioxidant therapy options including the use of vitamin D and pharmacologic therapies for hyperuricemia are discussed. Finally, we review some new therapy options in diabetic nephropathy including antidiabetic agents (noninsulin dependent), plant antioxidants, and food components as alternative antioxidant therapies.

## 1. Introduction

In chronic kidney disease both chronic and recurring acute inflammation are frequent. Underlying diseases, like autoimmune diseases, medication, uremic toxins, infections, and hemodialysis therapy are causal. The immune cells involved in those inflammatory processes produce free radicals in form of reactive nitrogen and reactive oxygen species. Overall, an imbalance between those free radicals and the available antioxidant capacity exists in chronic kidney disease (for review see Small et al., 2012, and Tucker et al., 2015, [1, 2]). Chronic kidney disease (CKD) denotes the presence of structural and/or functional abnormalities of the kidneys, with or without a reduction in glomerular filtration rate, with implications for health, lasting for more than three months [3]. The global prevalence of CKD in adults over 20 years of age was around 10% in men and 12% in women in a recent analysis [4]. The causes underlying CKD in a given population differ depending on ethnicity, region [5, 6], and age [7]. In children, congenital and hereditary disorders predominate. To this group belong cystic kidney diseases and obstructive uropathy. In adults, for example, in the United States the leading causes for CKD resulting in end-stage renal disease are diabetic nephropathy (type 2 diabetes mellitus accounts for around 30%, type 1 for around 6%), vascular diseases (like hypertension and ischemic renal disease) accounting for around 25%, glomerular diseases (including focal segmental glomerulosclerosis) accounting for around 18%, renal carcinoma, cystic diseases and tubulointerstitial disease [8].

CKD is characterized by a gradual loss of kidney function. It progresses through an initial lesion, the occurrence of repair mechanisms in which nephrons are lost, and the increase of activity of remaining nephrons that may be detrimental for nephron function. This disturbance frequently shows a pattern characterized by reduced glomerular filtration, disturbed salt and water balance, and loss of endocrine functions [9]. The development of fibrosis in the glomeruli and in the tubulointerstitial space is considered as common pathological alteration in CKD [10].

CKD is significantly linked to premature cardiovascular disease development. At the same time, cardiovascular disease (CVD) is the most common cause of death in CKD [11–13].

Underlying mechanisms comprise traditional cardiovascular risk factors that are common also in CKD patients like advanced age, hypertension, and diabetes mellitus. But since those traditional risk factors do not sufficiently account for the high cardiovascular risk in CKD CKD-associated risk factors have received much attention. The latter include malnutrition, inflammation, uremic toxins, proteinuria, bone and mineral metabolism abnormalities, persistent neurohormonal activation, and oxidative stress [14–16]. Currently, the following oxidative stress related mechanisms are thought to be especially important for the pathogenesis of CVD in CKD: protein-bound uremic toxins initiating oxidative stress-inflammation-fibrosis processes [16, 17], advanced glycation end products resulting in receptor-mediated and receptor-independent increase of oxidative stress, inflammation and vascular damage [18], chronic activation of the renin-angiotensin-aldosterone and sympathetic nervous system resulting in, also, oxidative stress-inflammation-fibrosis processes [19], and activation of the innate immune system leading to microinflammation and vascular dysfunction [20]. Mitochondrial dysfunction, causing increased oxidative stress and ATP depletion, is gaining attention in CKD and is discussed more in depth further down in this review.

Systemic oxidative stress is proposed to play a central role not only in the pathogenesis of CVD but also in kidney function decline and premature aging in CKD. Recent excellent reviews provided detailed overview over the current knowledge about the underlying molecular mechanisms and possible therapeutic interventions [21–23]. Especially in end-stage renal disease evidence-based therapeutic strategies to improve survival are limited (for review see [24]). The latest Cochrane review about “antioxidants for chronic kidney disease” in 2012 stated that “antioxidant therapy in CKD does not reduce the risk of cardiovascular and all-cause death” but “it is possible that some benefit may be present, particularly in those on dialysis” [25]. Antioxidant interventions in CKD, employing either antioxidant substances, substances that possess antioxidant effects among their mechanisms of action, or lifestyle interventions have been reviewed in depths recently: bardoxolone methyl [26], N-acetylcysteine [27, 28], vitamin E [27, 29], statins [30], renin-angiotensin-aldosterone system interventions [19, 31], interventions targeting gut-derived endotoxins and uremic toxins [16, 32, 33], and exercise training [34]. Selected substances and interventions with mechanistic and clinical information are given as supplementary material (see Supplementary Table 1 in Supplementary Material available online at http://dx.doi.org/10.1155/2016/6043601).

Nevertheless, the causal connection of oxidative stress to the genesis of cardiovascular disease in chronic kidney disease has also been controversially discussed [35, 36]. Those discussions were based on the one hand on the inconclusive results concerning the connection between supposed markers of oxidative stress and cardiovascular events and mortality in clinical studies [35, 36]. On the other hand, the up to date limited success of antioxidant therapies on cardiovascular outcomes in CKD patients but also in other populations demands a more differentiated view on oxidative stress-related pathogenic concepts and asks for new therapeutic approaches [37].

Our review therefore focuses on selected recent aspects in the discussion of pathogenic concepts and therapeutic approaches to oxidative stress in chronic kidney disease.

## 2. Mitochondrial Alterations in Chronic Kidney Injury and CKD

### 2.1. Mitochondrial Alterations in Chronic Kidney Injury

Reactive oxygen species (ROS) production has been clearly associated with the development of CKD and mitochondria are among the major ROS sources in renal diseases (reviewed in [38]). In fact, it has been found in several studies that oxidative stress in CKD patients enhances as the disease progresses [39–41]. Mitochondria are important in mammalian cells since these organelles supply all the necessary biological energy derived from the breakdown of carbohydrates and fatty acids, which is converted to adenosine triphosphate (ATP) via the process of oxidative phosphorylation. However, mitochondria also participate in cellular mechanisms associated with cell damage and cell death signaling [42–44]. The high energy demands of the kidney and other organs as brain and heart depend heavily on functional mitochondria. The kidney cells, particularly the mitochondria-rich proximal tubule epithelial cells, have high ATP requirements to facilitate active reabsorption of macromolecules [45–47]. About 80% of the oxygen consumed for ATP production supports active sodium transport while the basal metabolic rate accounts for only 15–20% of the oxygen consumption rate [48]. Mitochondrial content is closely regulated by mitochondrial biogenesis (the increase in the number of preexisting mitochondria) and mitophagy (the removal of damaged or dysfunctional mitochondria through autophagy). Both processes act to maintain mitochondrial homeostasis since mitochondrial biogenesis increases net mitochondrial mass to preserve mitochondrial functions by compensating for loss of damaged mitochondria by mitophagy [49–51]. Although mitochondria have their own DNA (mtDNA) most of the proteins that localize in mitochondrial membranes are nuclear gene products. Mitochondrial biogenesis involves the coordinated participation and expression of the genes localized in mitochondria and nuclei. Nuclear respiratory factors 1 and 2 (NRF1 and NRF2) are nuclear-encoded transcription factors that act on the nuclear genes coding for constituent subunits of the oxidative phosphorylation system and also regulate the expression of many other genes involved in mtDNA replication [52].

Peroxisome proliferator-activated receptor gamma (PPAR*γ*) coactivator-1 alpha (PGC-1*α*) is a nuclear-encoded transcriptional coactivator that regulates the expression of nuclear-encoded mitochondrial proteins, including NRF1 and NRF2 [51, 53]. PGC-1*α* is predominantly expressed in proximal tubules, indicating the effectiveness of PGC-1*α* in proximal tubular homeostasis [54]. Enforced overexpression of PGC-1*α* in cultured proximal tubular cells increased mitochondrial number, respiratory capacity, ATP concentration, and mitochondrial proteins [55].

Autophagy, derived from the Greek word meaning “self-eating,” is a catabolic pathway involving the degradation of cellular components by the hydrolases of lysosomes to maintain homeostasis and cell integrity [56, 57]. Dysregulated autophagy has been linked to many human pathophysiologies. Accumulating body of evidence implicates that autophagy regulates many critical aspects of normal and disease conditions in the kidney [44, 58].

Imbalance between mitochondrial biogenesis and the mitophagy process results in progressive development of numerous pathologic conditions associated with mitochondrial dysfunction characterized by the increase of mitochondrial ROS production, mitochondrial permeability transition (MPT) pore opening, and apoptosis [44, 59, 60].

It has been widely demonstrated that mitochondrial morphology plays an important role in cellular functions and that it is affected by the occurrence and rates of fission and fusion events [61]. Fission is the division of mitochondria within a cell to form separate mitochondrial compartments, while fusion is the merging of two or more mitochondria to form a single compartment. Dynamin-related protein (Drp-1) and mitochondrial fission protein 1 (Fis1) promote mitochondrial fission to create new mitochondria and to remove damaged mitochondria when cells are under stress [62, 63]. Mitochondrial fusion involves fusion of both the outer mitochondrial membrane and inner mitochondrial membrane, a process depending on mitofusin-1 (Mfn1), mitofusin-2 (Mfn2), and OPA1 (a dynamin-related protein with GTPase activity) [64]. It has been observed that defects in these proteins are closely related with alterations in both mitochondrial function and shape and as a consequence are associated with human diseases. For example, loss of function with Mfn2 mutation is related to Charcot-Marie-Tooth disease type 2A while OPA1 mutation is related to optic atrophy [65]. Until recently it has been appreciated that the equilibrium between fission and fusion events is important for mitochondrial function and distribution and, therefore, is pivotal for cell survival and health of the organism. Disturbances on this balance have a fundamental role in mitochondrial fragmentation and dysfunction [44, 65] and have been involved in a number of biological processes including cell division, apoptosis, autophagy, and metabolism. During cell injury, the equilibrium between mitochondrial fusion and fission shifts to mitochondrial fission and mitochondrial fragmentation occurs. This results in alterations in mitochondrial structure and morphology [66]. These changes might thereby contribute to kidney disease [67, 68].  Table 1 shows a list of markers used to evaluate mitochondrial biogenesis, mitochondrial dynamics (fission and fusion), and mitophagy.

### 2.2. Mitochondrial Alterations in CKD

Kidney cells contain abundant mitochondria, therefore mitochondrial dysfunction has a fundamental role in the development of kidney diseases. Many reports indicate the role of mitochondria in progression of CKD (reviewed in [69]). In the rat model of 5/6 nephrectomy Nath et al. [70] reported an increase in the rate of oxygen consumption in surviving nephrons and Fedorova et al. [71] observed in renal cortex of the same model a decrease in the expression in medium-chain acyl-coenzyme A dehydrogenase (MCAD) and cytochrome c oxidase subunit IV (COXIV) proteins as well as in the copy number of mtDNA. Sun et al. [72], using electron microscopy and confocal microscopy, reported alterations in mitochondrial structure and mitochondrial fragmentation in apoptotic tubular cells in kidneys from diabetic mice. Additionally, they reported cytochrome c release associated with apoptotic processes in tubular cells exposed to high glucose. These data reveal an association between mitochondrial dynamics and apoptosis in the progression of diabetic nephropathy. In fact, it is well known that oxidative stress induces apoptosis, a key process for the loss of functional tissue in CKD (reviewed in [1]). In this context, Daehn et al. [73] showed that apoptosis of podocytes in mice with glomeruloesclerosis secondary to adriamycin was associated with mitochondrial oxidative stress. Scavenging of ROS-derived mitochondria was able to protect podocytes and prevent renal failure and glomerulosclerosis. Furthermore, Chen et al. [74] showed that the protective effect of the antifibrotic drug pirfenidone of tubulointerstitial damage in 5/6 nephrectomized rats was associated with prevention of mitochondrial damage and with the decrease in tubular cell apoptosis and oxidative stress.

On the other hand, Wang et al. [75] found mitochondrial fission and fragmentation in renal cells in a conditional gene knock-out and knock-in mouse model in response to Drp-1 phosphorylation and activation by Rho-associated coiled coil-containing protein kinase 1 (ROCK1). Mitochondrial cytopathies (MC) are inherited mtDNA or nuclear DNA (nDNA) mutations in genes that affect mitochondrial functions. A single cell contains hundreds of mtDNA copies and, therefore, as a consequence of fission and fusion processes, the normal mtDNA may mix with mutant mtDNA. When the amount of mutant mtDNA copies surpasses the basal level a cell dysfunction occurs [69]. These alterations in kidneys result in focal segmental glomerular sclerosis (FSGS), tubular defects [69], cystic kidney disease [76–78], and renal carcinoma [79, 80].

Genetic defects connected to renal diseases include tRNA-LEU mutations (e.g., 3,243 A>G point mutation). So, in coenzyme Q10 (CoQ10) deficiency mutations of COQ1-PDSS2, COQ2, COQ6, and COQ9 have been reported. For impaired complex III assembly a mutation in BCS1L has been described and also a mutation in COX10 was reported leading to complex IV inactivation [69]. Mitochondria are also involved in epithelial to mesenchymal transition of renal tubular epithelial cells, a phenotypic conversion that contributes to the pathogenesis of renal interstitial fibrosis [69, 81]. Renal biopsies from patients showing simultaneously mitochondrial myopathy, encephalopathy, lactic acidosis, and stroke-like episodes (MELAS) syndrome and FSGS often show dysmorphic mitochondria in podocytes (epithelial cells with interdigitated foot processes that surround the glomerular capillaries) and effacement of podocyte foot processes.

Based on the fact that mtDNA mutations in podocytes were associated with FSGS in two children, Güçer et al. [82] postulated that podocytes with atypical mitochondria have a role in the development of glomerular diseases. FSGS is associated with genetic alterations and is characterized by altered mitochondria in podocytes and podocytes effacement [69]. Studies in mice subjected to aldosterone-induced renal injury show a decrease in mtDNA copy number, loss of mitochondrial membrane potential (Δ*ψ*_m_), drop of ATP production, and oxidative stress [83, 84]. These changes occur before proteinuria and podocyte process fusion can be observed. Podocyte foot process effacement also has been observed under high glucose conditions, probably through phosphorylation of Drp-1 by ROCK1 [75]. Mitochondrial alterations are also observed in arteriolar hyalinosis [85] as well as in steroid-resistant nephrotic syndrome [86]. So, it is clear that alterations in mtDNA may induce alterations in microvasculature and in podocytes, which are fatal insults to the kidney. Studies in peripheral blood mononuclear cells of CKD patients receiving peritoneal dialysis show decreased expression of NRF1 and PGC1-*α* and of several PGC1-*α* downstream target genes as mitochondrial transcription factor A (TFAM), cytochrome c oxidase subunit 6C (COX6C), cytochrome c oxidase subunit 7C (COX7C), mitochondrial Hinge protein ubiquinol-cytochrome C reductase Hinge gene (UQCRH), and MCAD [87]. In addition, Dugan et al. [88] found decreased protein and mRNA levels of PGC1*α* in kidneys of diabetic mice, which were prevented by the treatment of mice with the adenosine monophosphate-activated protein kinase (AMPK) activator 5-aminoimidazole-4-carboxamide-1-*β*-D-ribofuranoside (AICAR). Moreover, Granata et al. [89] reported an increase in ROS production, DNA oxidative damage and mitochondrial cytochrome c oxidase subunit 1 (COX1) expression, upregulation of 11 genes related to the oxidative phosphorylation system (ATP50, ATP51, and ATP5J: components of the ATP synthase complex V, NDUFS5, NDUFA6, NDUFA1, and NDUFB1: subunits of mitochondrial complex I, COX6C/COX7C: subunits of mitochondrial complex IV, and UQCRH and UQCRB: subunits of mitochondrial complex III), and a decrease in complex IV activity in peripheral blood mononuclear cells of CKD patients receiving hemodialysis. Granata et al. [89] concluded that CKD patients receiving hemodialysis had an impaired mitochondrial respiratory system.

The mitochondrial function at different stages of CKD remains to be fully studied. It has been found that, at early stages of 5/6 nephrectomy (24 h), renal dysfunction and mitochondrial oxidative stress are associated with decreased mitochondrial adenosine diphosphate induced respiration and low activity of respiratory complexes I and V. The mitochondrial alterations were prevented by the administration of the antioxidant curcumin [Aparicio-Trejo et al., manuscript submitted]. In contrast, it has been found on day 30 after 5/6 nephrectomy that renal mitochondrial bioenergetics was unaltered [90]. These data suggest that mitochondrial alterations are not similar along the development of CKD.

Many evidences indicate that mitochondrial dysfunction may be involved in the pathophysiology of kidney diseases although the mechanism responsible for the changes of mitochondrial dynamics under disease conditions is largely unknown. Future experiments should be directed to unravel these mechanisms as well as to design strategies to attenuate renal diseases using as a target the attenuation of mitochondrial alterations [91]. In addition, more studies are needed to evaluate the time-course changes of mitochondrial parameters along the development of CKD to establish the kinetic of these changes.

### 2.3. Cardiovascular Disease, Oxidative Stress, and Mitochondrial Deregulation

**Figure 1:**
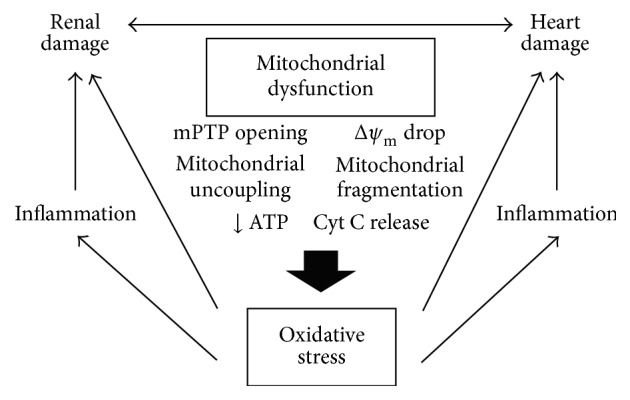
Integrative scheme of the mechanisms that cause kidney and heart damage secondary to mitochondrial dysfunction. Mitochondrial dysfunction represented by mitochondrial permeability transition pore opening, mitochondrial uncoupling/fragmentation, mitochondrial membrane potential loss, cytochrome C release, and decreased ATP synthesis, among other mitochondrial alterations, causes oxidative stress that leads to inflammatory state. Both conditions result in renal and cardiac damage that often occurs at the same time and establishes a intercommunication through hemodynamic and nonhemodynamic mechanisms.

Mitochondrial alterations also have been found in hearts when renal function declines (Figure 1). Using an experimental model of cardiorenal syndrome, Sumida et al. [92] reported mitochondrial fragmentation, increased dynamin-related protein 1 (DRP-1) expression, apoptosis, and cardiomyocyte dysfunction in hearts of mice subjected to bilateral renal ischemia reperfusion. The inhibition of DRP-1 attenuated significantly the changes observed in the heart. The study of Hernández-Reséndiz et al. [93] is another example of interrelation between kidney and heart. Rats with 5/6 nephrectomy exhibited elevated systolic blood pressure, proteinuria, cardiac dysfunction, oxidative stress, activation of apoptotic mitochondrial pathway, and alterations in cardiac mitochondrial integrity (inability to retain calcium and fall in transmembrane potential). All these cardiac, systemic, and mitochondrial alterations were prevented by the administration of curcumin. Moreover, Taylor et al. [94] also demonstrated a cross-talk between the kidneys and the heart using the 5/6 nephrectomy model in rats. They found that state 4 respiration was enhanced. In addition, after ischemia reperfusion uremic mitochondria showed a significant increase in state 4 respiration and reduction in respiratory control ratio and uremic cardiomyocytes were more vulnerable to H_2_O_2_-induced stress.

## 3. Hyperuricemia, Oxidative Stress, and CKD

Uric acid is the product of purine metabolism in primates including man and is an essential antioxidant for these organisms, for they have lost uricase activity during evolution. It has been suggested that this particular antioxidant system could have replaced the lost capability for vitamin C synthesis, therefore, allowing humans to evolve as uric acid increased longevity [95] and provided neuroprotection [96]. Due to the similarity of uric acid to the caffeine molecule, that characteristic also enabled it to act as a mental stimulant [97], therefore, providing an advantage for the development of human intelligence. Another benefit of an increased concentration of uric acid in humans is that it stimulates the activity of the renin-angiotensin system. This effect facilitated the maintenance of blood pressure during the evolution to bipedalism under the low sodium diets, prevalent during human evolution [98]. Humans are prone to conserve uric acid, and its renal excretion is limited to 8–10% of the filtered load. Like other antioxidants, uric acid may assume prooxidant roles [99] and this effect could partially explain why epidemiologic studies have associated hyperuricemia with hypertension, metabolic syndrome, and chronic kidney disease [100–106].

Hyperuricemia has been arbitrarily defined in men >7 mg/dL and in premenopausal women >6.5 mg/dL. However, serum uric acid concentrations are greatly influenced by diet, mainly by red meat, seafood, alcohol, and fructose consumption. Therefore, as these dietary items became more affordable for the general population, the concentrations of uric acid “considered” normal have been progressively increasing from the beginning of the twentieth century [107].

Despite its described role as an antioxidant, the first response to uric acid exposure is a rapidly increase in oxidative stress in endothelial cells, proximal tubule epithelial cells, mesangial cells, vascular smooth muscle cells, hepatocytes, and adipocytes [108–115]. Although the effect of hyperuricemia on renal podocytes has not been directly addressed it is likely that uric acid may have effects on these cells similar to other renal cell types, such as the activation of NALP3 inflammasome [116] and NOX-4 [117]. These studies showed that it is the uric acid which enters into cells that is responsible for increasing intracellular oxidative stress through a mechanism that includes the activation of NADPH oxidase [118]. Interestingly, some of the deleterious effects induced by hyperuricemia are similar to those associated with increased oxidative stress such as reduced nitric oxide bioavailability and endothelial dysfunction, vascular hypertrophy, and inflammation and activation of the renin-angiotensin system [119]. The harmful effects of mild hyperuricemia on kidney function have been documented. Hyperuricemic nephropathy is induced in the laboratory rat by the inhibition of liver uricase. This maneuver induced hypertension, renal vasoconstriction, glomerular hypertension, arteriolopathy, tubulointerstitial fibrosis, and inflammatory infiltration. In this model, the renal and vascular damage was mediated by soluble uric acid, in contrast to gouty nephropathy which is mediated by uric acid crystal deposition [120]. The role of oxidative stress as pathogenic mechanism induced by uric acid was further documented, for the treatment of hyperuricemic rats with an antioxidant prevented hypertension as well as the renal functional and structural alterations induced by hyperuricemia [117]. In addition, deposition of uric acid crystals in proximal tubule cells is also associated with increased oxidative stress and activation of the NRLP3 inflammasome [116].

Since hyperuricemia strongly correlates with other metabolic factors (obesity, dyslipidemia, and insulin resistance) its role as a true cardiovascular risk factor in healthy individuals is still under discussion [121]. But increased serum uric acid levels have been found to be an independent risk factor for hypertension, diabetes, chronic kidney disease, and congestive heart failure; moreover, hyperuricemia has a predictive value for the development of vascular and kidney disease in these conditions [122–125].

In CKD patients, a 6-year follow-up found that hyperuricemia showed a J-shaped independent association with all-cause mortality; therefore both abnormally low and high levels of serum uric acid increased the mortality risk [126]. Interestingly, low concentration of serum uric acid is a consequence of malnutrition and high comorbidity burden; this suggests that systemic oxidative stress may be a causative factor for increasing the mortality in these patients [127]. In hyperuricemic hemodialysis patients, reduction of serum uric acid with febuxostat decreased oxidative stress and improved endothelial dysfunction [128].

Hyperuricemia increases as GFR declines in CKD patients. Moreover, gout increases the risk of cardiovascular events and all-cause mortality in hemodialysis patients [129]. Interestingly, it was shown in renal biopsies of CKD patients that hyperuricemia was associated with vascular alterations consistent with arteriolopathy, a lesion frequently observed in experimental studies [130]. Nevertheless, some others have reported nonsignificant associations between hyperuricemia and progression of kidney disease [131, 132]. Accordingly, there is an increasing interest in determining the efficacy of treating hyperuricemia in CKD patients. The use of allopurinol, febuxostat, and topiroxostat therapies decreased serum uric acid and slowed the progression of CKD in five small trials [133–137]. As the debate continues about the role of uric acid as a causative factor or only a marker of renal dysfunction [138], the need for clinical studies including a greater number of patients is evident. In this regard, currently a major NIH trial, including six academic centers, is ongoing to determine if lowering uric acid with allopurinol may provide benefit in type 1 diabetic subjects with early evidence of renal disease (PEARL) [139].

Whether hyperuricemia could confer risk for developing CKD in normal subjects is also a relevant issue. A recent meta-analysis, which included more than 190,000 non-CKD individuals, found that hyperuricemia is an independent predictor for the new-onset of CKD [140]. In addition, the treatment of hyperuricemia provides benefit for controlling risk factors associated with the development of CKD. Thus, in adolescents with early onset of essential hypertension and obese subjects with prehypertension, allopurinol treatment significantly reduced systolic and diastolic blood pressure [141, 142].

In conclusion, the role of hyperuricemia as a true risk factor for the development of CKD is still under debate. The discussion is complicated by the fact that the noxious effects of uric acid occur inside the cell; thus, serum uric acid might not fully reflect this phenomenon. More studies to disclose the impact of hyperuricemia in the pathogenesis of CKD are warranted.

## 4. Antioxidant Potential of Vitamin D in Chronic Kidney Disease

Chronic kidney disease (CKD) is accompanied by reduced plasma concentrations of 25-dihydroxyvitamin D and 1,25-dihydroxyvitamin D to varying extent.

Vitamin D is taken up with the diet or produced in the skin after exposure to ultraviolet rays of sunlight [143]. In the liver vitamin D is hydroxylated to 25-hydroxyvitamin D probably by the cytochrome P-450 CYP2R1 [144]. A further hydroxylation step to 1,25-dihydroxyvitamin D is achieved through 1-alpha-hydroxylase (cytochrome P-450 27B1) activity [145]. This takes place in the kidney and in a multitude of extrarenal cells. Renal 1,25-dihydroxyvitamin D production mainly serves endocrine purposes like regulation of bone and calcium-phosphate metabolism. Vitamin D effects on the immune system or cell proliferation are supposed to be mainly due to auto- and paracrine actions of 1,25-dihydroxyvitamin D produced in extrarenal cells [146]. The effects of vitamin D are mediated to a large extent through binding to a nuclear receptor, the vitamin D receptor [147].

Numerous groups have reported important insights about the connection between the vitamin D receptor and oxidative stress or oxidative damage. In their vitamin D receptor knock-out model Kállay et al. showed that a significant increase of oxidative DNA damage occurred with complete loss of vitamin D receptor [148]. Aortic smooth muscle cells from vitamin D receptor knock-out mice showed increased NADPH oxidase-dependent superoxide anion production [149]. Interestingly, curcumin that shows a variety of antioxidant effects (for review see [150]) was also shown to be a vitamin D receptor ligand and increased the expression of vitamin D responsive element containing genes [151, 152].

Another important molecular link between the vitamin D system and oxidative stress or oxidative damage is the alpha klotho protein that was originally identified to be an antiaging factor [153]. The kidney is an important source of circulating alpha klotho and both kidney and circulating alpha klotho amount are reduced in CKD [154–156]. Circulating alpha klotho concentrations can be increased by vitamin D receptor agonists through vitamin D receptor-mediated gene expression [154, 157]. Antioxidant effects of alpha klotho have been widely described. Alpha klotho protected lung and lung epithelial cells through increased antioxidant capacity while alpha klotho deficiency in acute kidney injury increased oxidative damage to these cells due to decreased antioxidant capacity [158, 159]. In aortic smooth muscle cells alpha klotho upregulated antioxidant enzymes and glutathione [160]. In retinal pigment epithelium the production of reactive oxygen species was reduced by alpha klotho [161]. Together, alpha klotho and 1,25-dihydroxyvitamin D promote antioxidation (for review see [162]).

In animal models vitamin D deficiency resulted in oxidative and nitrosative stress and was suggested to enhance contrast media nephrotoxicity by an oxidative stress-related mechanism [163–165]. In humans with chronic hepatitis vitamin D insufficiency was associated with increased global oxidative stress markers [166].

In cell and animal studies protective effects of vitamin D against oxidative stress or oxidative damage were reported by a multitude of research groups. In skin cells 1,25-dihydroxyvitamin D treatment was able to reduced UV-induced DNA damage [167]. The pretreatment of human umbilical vein endothelial cells with 1,25-dihydroxyvitamin D significantly reduced acetoacetate-induced oxidative stress and inhibited superoxide anion generation [168, 169]. Also, gene ontology analysis of vitamin D receptor activation in human vascular smooth muscle cells revealed modulation of genes related to antioxidant activity [170].

In CKD proteinuria (>500 mg/day) seems to be causally connected to CKD progression. Alterations in kidney podocyte function or loss of podocytes can contribute to proteinuria. Effects of vitamin D on podocyte function were extensively studied in cellular and animal models [171–173]. In a rat model of diabetic nephropathy Song et al. found an amelioration of podocyte injury by calcitriol [174]. Garsen et al. showed reduced proteinuria by 1,25-dihydroxyvitamin D treatment connected to reduced heparanase expression in podocytes [175]. In two animal models of kidney injury 1,25-dihydroxyvitamin D reduced podocyte urokinase receptor expression and proteinuria [176]. In a mouse model of HIV-associated nephropathy a downregulation of vitamin D receptor in renal tissue was observed, and HIV also* in vitro* downregulated vitamin D receptor expression in podocytes. In parallel, reactive oxygen species generation and DNA damage were upregulated, effects that could be reduced by vitamin D receptor agonist treatment [177].

Considering the abovementioned mechanisms, it is interesting now to have a look at vitamin D interventions in CKD. Several groups investigated vitamin D effects on markers of oxidative stress or enzymes with pro- or antioxidant activity. Most studies investigating vitamin D receptor agonist effects were performed with paricalcitol. Husain et al. reported that in uremic rats paricalcitol treatment reduced uremia-induced cardiac NADPH oxidase upregulation, increased uremia-impaired cardiac glutathione content, and improved the uremia-dependent reduction of cardiac copper/zinc superoxide dismutase activity [178]. Also in a uremic rat model, paricalcitol improved the uremia-dependent downregulation of renal copper/zinc superoxide dismutase protein and reduced uremia-induced oxidative stress in the kidney [179]. Another vitamin D receptor agonist, doxercalciferol, decreased inflammation and oxidative stress in a dietary fat-induced renal disease mouse model [180]. In humans direct investigation of vitamin D effects on tissue-specific oxidative stress is less feasible. Therefore in clinical studies mainly global parameters of oxidative stress or damage were investigated. One uncontrolled study with paricalcitol in hemodialysis patients reported a significant reduction of global parameters of oxidative stress and an increase of parameters of antioxidant capacity [181]. A recent randomized controlled trial of effects of paricalcitol in CKD stages 3 and 4 over 3 months did not show a significant influence on global markers of inflammation or oxidative stress [182].

Vitamin D effects on oxidative stress in CKD are thought to be related to effects on uremia-dependent changes of inflammatory state, antiproliferation, and immune function and hence to nonclassical vitamin D actions. It was therefore considered as reasonable to improve 25-dihydroxyvitamin D supply for extrarenal production of 1,25-dihydroxyvitamin D [183]. The results obtained from clinical studies testing this approach were conflicting. Markers of oxidative stress were not assessed directly but markers of immune cell activation and inflammation might be regarded as indicators. In an 8-week and a 12-week randomized controlled trial with cholecalciferol in hemodialysis patients no significant effects on inflammatory markers and cytokines could be detected [184, 185]. In three uncontrolled trials in hemodialysis patients, cholecalciferol therapy between 8 weeks and 1 year caused significant reductions of inflammatory markers and cytokines [186–188]. In CKD patients not on hemodialysis treatment a randomized controlled trial over one year did not show a sustained effect on cytokines and markers of inflammation [189]. An uncontrolled trial with 28 weeks of cholecalciferol treatment showed a significant reduction of urinary transforming-growth-factor-beta 1 [190]. The varying results of cholecalciferol interventions in CKD might be due to differences in methodology. Also, yet unrecognized differences in patient characteristics could be involved.

The overall discrepancy between vitamin D effects on oxidative stress markers and inflammation between preclinical and clinical studies which applies to some extent to both cholecalciferol and vitamin D receptor agonists is probably based on the multitude of disturbances of vitamin D metabolism and signal transduction in CKD. Uremia-dependent impairment of 1,25-dihydroxyvitamin D production also in extrarenal cells, uremia-dependent impairment of 25-hydroxyvitamin D uptake and intracellular transport in those cells, the reduced expression of vitamin D receptor protein in CKD, and the disturbed interaction of vitamin D receptors with DNA in uremia can contribute to the lesser than expected effects of vitamin D therapy in CKD (for review of mechanisms see [191, 192]).

More research is needed in the future, to determine in individual CKD patients those factors that result in a positive response to vitamin D treatment with respect to oxidative stress or inflammation. Also, new treatment strategies, which specifically target disturbances of vitamin D metabolism in CKD, for example, the reduction of vitamin D receptor protein, might be promising.

## 5. Emerging Therapies for Diabetic Nephropathy

**Figure 2:**
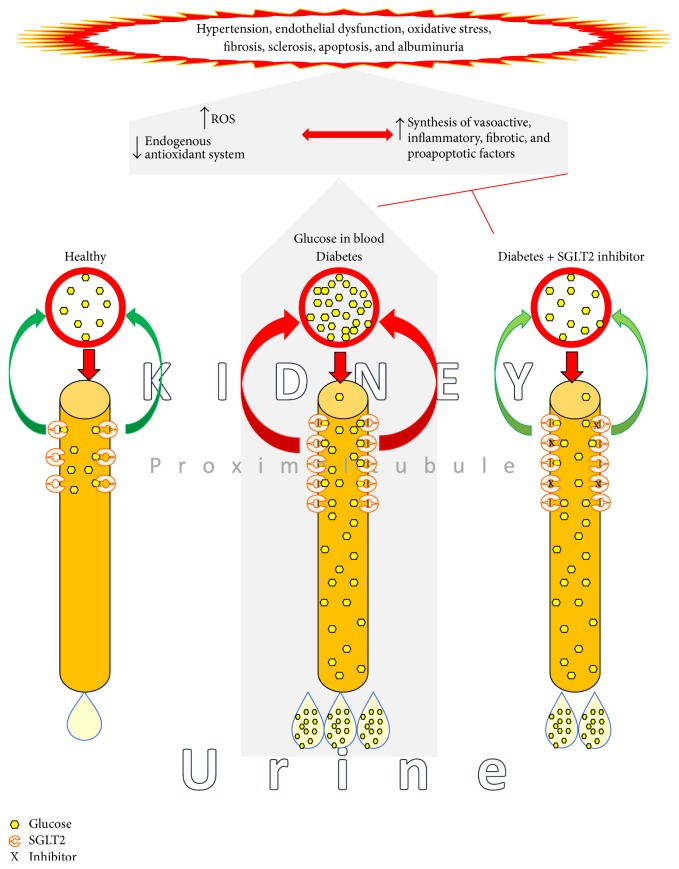
Role of sodium-glucose cotransporter 2 in blood glucose control in basal and hyperglycemic conditions and effects on blood glucose.

Diabetes mellitus (DM) is a metabolic disorder characterized by chronic hyperglycemia. The chronic exposure to high glucose concentrations damages certain tissues and organs like the kidneys, for example, by noninsulin dependent glucose uptake. Interactions of hemodynamic, metabolic, and humoral factors are all thought to be involved in the pathogenesis of diabetic nephropathy (DN) [193, 194]. Hyperglycemia activates prooxidant, profibrotic, and proinflammatory pathways leading to endothelial dysfunction, mesangial matrix accumulation, podocyte detachment and loss, glomerular basement membrane thickening, vacuolization in tubular epithelial cell, tubular atrophy, fibrosis, and tubulointerstitial inflammation (see Figure 2) [195–197].

The available antidiabetic agents have been developed to target one or more of the underlying defects or processes involved in DN. However, currently, therapies have not been fully effective, which makes it necessary to search for new therapeutic options for the management of this disease. Of these options, antioxidant-based therapies and inhibitors of sodium-glucose cotransporter 2 (SGLT2 inhibitors) are recent developments.

In basal condition, ninety percent of glucose filtered by the glomerulus is reabsorbed by the low-affinity/high capacity cotransporter SGLT2, which is expressed mainly on S1 and S2 segment of renal proximal tubules (Figure 2). However, during hyperglycemia the blood glucose concentrations are high, which is associated with an increased ability to reabsorb filtered glucose [198–201], due to an increase in SGLT2 expression [201–206]. This mechanism is counterproductive for the patient since glucose in plasma increases. Chronic exposure of renal cells to high glucose concentrations therefore causes a chronic vicious circle and is harmful (Figure 2).

SGLT2 inhibitors are a new class of drugs with a unique action mechanism that is insulin-independent and depends on plasma glucose and renal function. The use of SGLT2 inhibitors (dapagliflozin, canagliflozin, empagliflozin, and ipragliflozin) significantly reduces hyperglycemia, body weight, glycated hemoglobin (HbA_1C_), blood pressure (BP), hyperinsulinemia, inflammatory markers (interleukin-6 (IL-6), tumor necrosis factor *α* (TNF-*α*), monocyte chemotactic protein-1 (MCP-1), and C-reactive protein (CRP)), hyperfiltration, natriuresis, oxidative stress (OS), and glycosuria [202, 203, 207–210]. The therapeutic advantages of using SGLT2 inhibitors in clinical practice are summarized in Table 2.

Additional therapeutic benefits induced by SGLT2 inhibitors could be mediated by an indirect effect on blood glucose, which may be the most important mechanism associated with improvement of renal function and other complications related to DN (Table 2). These data suggest that SGLT2 inhibitors may have a renoprotective effect in diabetes. However, certain adverse events or potential risk related to increased glycosuria including a higher frequency of urinary infections, genital fungal infections, volume depletion, and a low risk of hypoglycemia have been described.

Recently, traditional, complementary, and alternative medicines are considered to cope with the mechanisms involved in the progression of DN, mainly against OS and hyperglycemia. The use of herbal medicinal plants especially those used in folk medicine for the treatment of DM is common in the world. Among these are foods commonly consumed or their derivatives such as garlic, curcumin, moringa, cinnamon, resveratrol, and sulforaphane.

Garlic* (Allium sativum) *is a common cooking spice used as a folk remedy, which has been experimentally described to have antidiabetic potential. The garlic extract showed a significant improvement in blood glucose, fasting plasma glucose (FPG), HbA_1C_, serum insulin levels, lipid peroxidation, total antioxidant level (TAL), catalase activity (CAT), urine, and serum biochemical parameters such as albumin, urea nitrogen, and creatinine compared to that of diabetic rats. Further, garlic supplemented diabetic rats showed less glomerular glycation, loss of microvilli of proximal tubules, extravasation of red blood cells, thickness of the glomerular basement membrane, and expression of VEGF and ERK-1 compared to diabetic rats, attenuating mesangial expansion and glomerulosclerosis [211–215]. The use of garlic derivatives including S-allyl cysteine and allicin has been found effective in lowering blood glucose levels, improving OS markers (CAT, superoxide dismutase (SOD), and glutathione) and protecting cell protein and cell membranes [216, 217]. Other studies reported that garlic downregulates expression of angiotensin II AT1 receptors (AT1) and receptor for advanced glycation end products (RAGE) in renal cortical glomeruli and tubules [214, 218].

Although experimental studies described antidiabetic effects of garlic, human studies are inconclusive but showed a compelling antioxidant effect. Aged garlic extract intake (3 g/day) did not affect blood glucose, HbA_1C_, or the lipid profile but did reduce levels of serum advanced glycation end products (AGEs) and lipid hydroperoxide in patients [219]. In contrast, other studies describe that garlic extract improves blood lipid profile, strengthens TAL, and decreased lipid peroxidation, BP, RAGE, and secretion of IL-1 and TNF in patients [220, 221].

Curcumin is a yellow pigment from* Curcuma longa*, commonly consumed as a flavor and coloring food. In experimental diabetes curcumin has been shown to improve blood glucose, HbA_1C_, lipid profile, serum creatinine, blood urea nitrogen (BUN), and kidney/body weight ratio and to significantly reduce blood concentrations of IL-6, MCP-1, TNF-*α*, and OS, which was evidenced by its effects on 8-hydroxy-2′-deoxyguanosine (8-OHdG), malondialdehyde (MDA), 3-nitrotyrosine (3-NT), GSH, and antioxidant enzymes levels (SOD, CAT, and heme oxygenase-1 (HO-1)) [222–227]. Curcumin decreased ROS production and apoptosis via dephosphorylation of caveolin-1 (cav-1) in kidneys and in podocytes* in vitro* [226].

Clinical studies report that curcumin can effectively prevent the prediabetes population from developing T2DM, attenuate proteinuria, and exert immunomodulatory effects on circulating concentrations of IL-1*β*, IL-4, IL-8, TGF, and VEGF [228–230]. In a recent study curcumin did not improve proteinuria, GFR, or lipid profile. However, curcumin attenuated lipid peroxidation and enhanced the antioxidant capacity in plasma [231]. On the other hand, the combination of garlic extract and curcumin decreased HbA_1C_, FPG, and 2-hour postprandial blood glucose but the treatment did not affect liver and kidney function [232].

It has been suggested that the beneficial effects induced by curcumin involve the downregulation of Wnt/*β*-catenin signaling as well as PKC-*α* and PKC-*β*1 activities, phosphorylated ERK1/2 in renal glomeruli, and enhanced nuclear translocation of Nrf2 and preservation of the activity of antioxidant enzymes [90, 233, 234]. Curcumin acts through normalizing the expression levels of various factors important during DN progression. This includes normalization of the levels of NOX4, p67phox, TGF-*β*, CTGF, osteopontin, vimentin, desmin, SREBP-1, iNOS, synaptopodin, connexin 43, erythropoietin, p300, and extracellular matrix proteins [227, 233, 234].

Moringa (*Moringa oleifera *L.) is the cultivated species of the genus* Moringa* of the family Moringaceae. The moringa extract demonstrated a beneficial effect on body weight, blood glucose concentration, renal function, lipid peroxidation, and activities of SOD, CAT, GST, and GSH in renal tissue, as well as TNF and IL1 concentrations in serum [235–239]. In addition, immunoglobulins (IgA, IgG), FPG, and HbA_1C_ were also decreased, and the histology of both kidney and pancreas were restored with moringa treatment [235].

Clinical studies showed that moringa significantly decreased FPG, hyperglycemia, total cholesterol, triglycerides, low-density lipoprotein- (LDL-) cholesterol, and VLDL-cholesterol [240–242]. Moreover, the data revealed significant increases in serum GPx and SOD, with decreases in MDA [243].

One of the most widely used spices in the food and beverage industry is cinnamon. The administration of cinnamon to diabetic rats decreased blood glucose and lipid peroxidation and improved lipid profile and GPx, SOD, and CAT activities in the kidney [244]. Cinnamon also protects the kidney by reducing glomerular expansion, eradicating hyaline casts, decreasing the tubular dilatations, and restoring nucleus and cytoplasm material of both glomerulus and Bowman's capsule [245, 246]. Procyanidin-B2, the active compound of cinnamon, inhibits* in vitro* and* in vivo* AGE formation and accumulation in diabetic kidney. Interestingly, procyanidin-B2 prevented the loss of expression of nephrin and podocin [247].

Cinnamon seems to have a moderate effect in reducing FPG, HbA_1C_, blood triglyceride levels, and OS markers in patients [248–250]. It has been described that the antidiabetic effect of cinnamon may be due to enhanced insulin receptor phosphorylation and the translocation of glucose transporter-4 (GLUT4) [251]. Another mechanism that explains the effects of cinnamon is an increase in the expression of peroxisome proliferator-activated receptor (PPAR), alpha and gamma receptors, thereby increasing insulin sensitivity [252].

Resveratrol (trans-3,4′,5-trihydroxystilbene), a polyphenolic compound naturally existing in grapes, has shown antioxidant activity. Resveratrol treatment ameliorates hyperglycemia, renal production of ROS, apoptosis, inflammation, and renal dysfunction in diabetes [253–258]. Moreover, resveratrol prevented the reduction in podocyte number and the disruption of both podocyte foot processes and basal infoldings [257]. In diabetic patients resveratrol significantly decreased BP, FBG, HbA_1C_, total cholesterol, and insulin resistance, while HDL was significantly increased compared to their baseline levels [259, 260].

The protective effects of resveratrol, with respect to cell apoptosis and ROS include activation of AMP-activated protein kinase (AMPK), silent information regulator T1 (SIRT1), and PPAR*γ* coactivator 1*α* (PGC-1*α*) and the consequent effects on its target molecules PPAR*α*-oestrogen-related receptor and the phosphatidylinositol-3 kinase-protein kinase B–O forkhead box 3a pathway in diabetes [256]. Also, antioxidant effects of resveratrol in diabetes are related to the Nrf2/Keap1 pathway and downstream regulatory proteins [257].

Recently, it was reported that resveratrol decreased mesangial cell proliferation, glomerular basement membrane thickness, fibrosis and the expression of plasminogen activator inhibitor (PAI-1), ICAM-1, protein kinase B (Akt), vascular endothelial growth factor (VEGF), and its type 2 receptor Flk-1 and inhibited the nuclear factor-kappa B (NF-*κ*B) [258–261]. This suggests that resveratrol may attenuate DN via the modulation of Akt/NF-*κ*B pathway and influences on angiogenesis.

Sulforaphane is an organosulfur compound obtained from crucifers vegetables such as broccoli, brussels sprouts, or cabbages. Sulforaphane exhibits antioxidant and antidiabetic properties in diabetes. Sulforaphane prevents diabetes-induced inflammation, renal dysfunction, and oxidative and nitrosative damage [262, 263]. In patients, sulforaphane decreased MDA, oxidized LDL-cholesterol, and oxidative stress index and there was a significant increase in TAL [264].

Some studies have shown that activation of Nrf2 by sulforaphane improved hyperglycemia, albuminuria, pathological alterations in the glomerulus, and oxidative damage and suppressed the expression of TGF-*β*1, FN, Col IV, and p21 both* in vivo* and in human renal mesangial cells [265]. Also, sulforaphane increased Nrf2 at protein and mRNA levels in kidney, which leads to a higher expression of NAD(P)H: Quinone Oxidoreductase 1 (NQO1), HO-1, SOD1, SOD2, and CAT at mRNA and protein levels [262, 263].

**Figure 3:**
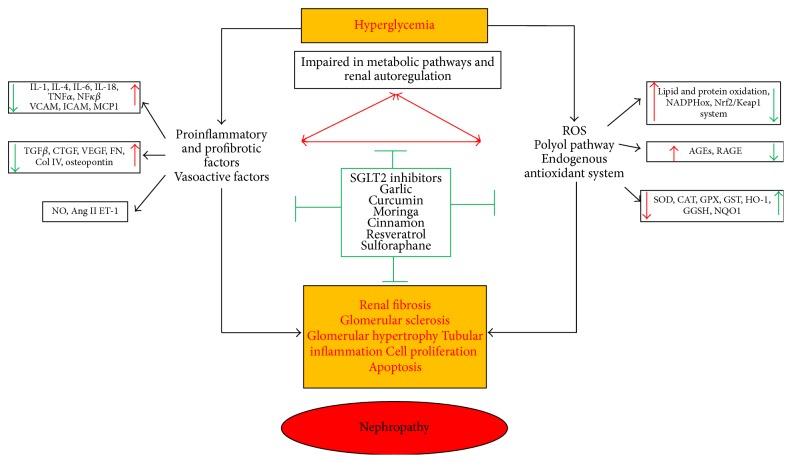
The pathophysiological mechanism of diabetic nephropathy and targets for emerging therapies. Advanced glycation end products (AGEs), endothelin-1 (ET-1), receptor for AGEs (RAGES), superoxide dismutase (SOD), catalase (CAT), nicotinamide adenine dinucleotide phosphate oxidase (NADPHox), glutathione (GSH), glutathione peroxidase (GPX), glutathione reductase (GR) and glutathione-s-transferase (GST), transforming growth factor-*β*1 (TGF-*β*1), tumor necrosis factor-*α* (TNF-*α*), reactive oxygen species (ROS), sodium-glucose cotransporter 2 (SGLT2), nuclear transcription factor-kappa-B (NF-*κ*B), vascular endothelial growth factor (VEGF), Monocyte Chemoattractant Protein (MCP-1), connective tissue growth factor (CTGF), fibronectin (FN), vascular cell adhesion molecule-1 (VCAM-1), intracellular adhesion molecule-1 (ICAM-1), heme oxygenase-1 (HO-1), NF-E2-related factor-2 (Nrf2), and collagen type IV (col IV).

All these studies suggest that those emerging therapies could be a therapeutic option in the combat of DN (see Figure 3). Although in experimental models the results have been promising, in a clinical setting they are still controversial. The discrepancy might be accounted for by differences in physiology between laboratory animals and humans but also by differences in the type of preparation and used concentrations; thus, the chemical composition of these preparations varies widely and the biological responses are different. Another possible reason for differences between clinical studies and experimental ones may be deficiencies in methodology and different treatment durations. Experimentally, the dose and the duration of medication can be exactly controlled; however in humans, sufficient control of these aspects can be challenging.

In conclusion, hyperglycemia and oxidative stress plays a key role in the progression of diabetic nephropathy (Figure 3). Emerging therapies achieve their beneficial effects through glycemic control and regulation of antioxidant status, suggesting them as attractive therapeutic alternatives (Figure 3). However, results from clinical trials have been inconclusive so far. Therefore, further studies are needed to ascertain whether the new alternative options offer benefits as first-line drugs or adjuvant medication in clinical practice.

## 6. Conclusions

Taken together, the knowledge about pathogenic aspects of oxidative stress for the progression and for complications in chronic kidney disease has significantly been extended. New therapeutic antioxidant approaches are available but are not yet satisfyingly developed and validated.

## Supplementary Material

Supplementary table 1 provides selected current antioxidant approaches together with the respective mechanistic and clinical information.

## Figures and Tables

**Table 1:** Markers used to evaluate mitochondrial biogenesis, mitochondrial dynamics, and mitophagy.

Mechanism	Marker	Function (site)
Mitochondrial biogenesis	Subunit 1 of mitochondrial NADH dehydrogenase mitochondrial (MT-ND1).	Subunit of NADH dehydrogenase (mitochondrial inner membrane).
	Mitochondrial transcription factor A (TFAM).	Activator of mitochondrial transcription (mitochondria).
	Nuclear respiratory factors 1 and 2 (NRF1, NRF2) and estrogen receptor alpha (ERR*α*).	Transcription factors for mitochondrial biogenesis (nuclei).
	Peroxisome proliferator-activated receptor gamma, coactivator 1 alpha (beta) (PGC1*α*, PGC1*β*).	Transcriptional coactivator that regulates the genes involved in mitochondrial biogenesis (nuclei).

Mitochondrial dynamics	Dynamin related protein 1 (Drp-1). Mitochondrial fission protein 1 (Fis1).	Mitochondrial fission.
	Sirtuin 3 (SIRT3).	Decreases mitochondrial fission.
	Optic atrophy 1 protein (OPA1). Mitofusin-1 (Mfn1) protein. Mitofusin-2 (Mfn2) protein.	Mitochondrial fusion.

Mitophagy	Pten-induced kinase 1 (PINK1).	PINK1 activity causes the parkin protein to bind to depolarized mitochondria to induce autophagy.
	Parkin protein.	Mediates the targeting of proteins for degradation.
	Mitochondrial E3. Ubiquitin ligase 1 (Mul1).	Activator of mitophagy.
	FoxO1/FoxO3 transcription factors.	Activators of mitophagy.

**Table 2:** Long term effects of SGLT2 inhibitors as approved glucose-lowering agents.

Inhibitor	Diabetes	Effects	References
Canagliflozin	Type 2	Reduces GFR, HbA_1C_, BW, BP, FPG.	[266, 267]
Dapagliflozin	Type 2	Reduces GFR, BP, BW, HbA_1C_, albumin and stabilizes insulin dosing.	[268–272]
Empagliflozin	Type 1 Type 2	Reduces GFR, plasma NO, HbA_1C_, arterial stiffness, heart failure hospitalization, cardiovascular death.	[207, 273–275]
Ipragliflozin	Type 2	Reduces HbA_1C_, BW, FPG and improves liver function and lipid profile.	[276–278]

Glycated haemoglobin, HbA_1C_, glomerular filtration rate, GFR, fasting plasma glucose, FPG, body weight, BW, blood pressure, BP, and nitric oxide, NO.
