# Supplementary material for: New Pathogenic Concepts and Therapeutic Approaches to Oxidative Stress in Chronic Kidney Disease
Source: Oxid Med Cell Longev. 2016 Jun 27;2016:6043601. doi: 10.1155/2016/6043601 (PMC4939360; doi:10.1155/2016/6043601)
Supplement: Supplementary file 1 — Supplementary table 1 provides selected current antioxidant approaches together with the respective mechanistic and clinical information. [file 6043601.f1.pdf]

Supplementary table 1 Selected antioxidant approaches with example substances and interventions in chronic kidney disease.

Approach	Example substance/ intervention	Mechanism of action	Ref.	Clinical effects	Ref.
nuclear factor erythroid 2-related factor (Nrf2) activation	*bardoxolone methyl*	• interaction with the Nrf2 repressor Keap1 (Kelch-like ECH-associated protein 1) -> facilitation of nuclear translocation of Nrf2 -> upregulation of antioxidant and cytoprotective target genes	[1]	• in patients with advanced CKD and type II diabetes mellitus improvement of estimated GFR<br><br>• in patients with CKD stage 4 and type II diabetes mellitus no reduction of end-stage renal disease risk or of cardiovascular death; increased rate of cardiovascular events	[3]<br><br><br>[4]
	dh404 (bardoxolone analog)	• as above,<br>• dose-dependency (lower concentrations activate Nrf2 and target genes; higher concentrations suppress Nrf2 system)	[2]		
thiol (sulfhydryl) containing antioxidants	*N-acetylcysteine*	• free radical scavenger<br><br>• maintenance of intracelluar glutathione<br><br>• post-translational modification of protein cysteine residues *in vivo*	[5]<br><br>[6]<br><br>[7]	• in hemodialysis patients reduction of composite cardiovascular endpoints; no reduction of mortality<br><br>• in peritoneal dialysis patients reduction of interleukin 6	[8]<br><br><br>[9]
vitamin E supplementation	*alpha-tocopherol*	• incorporation into membrane bilayers -> interruption of lipid peroxidation chain reactions by free radical scavenging	[10]	• in hemodialysis patients with prevalent cardiovascular disease reduction of myocardial infarction and composite CVD endpoints, no effect on total mortality	[11]
	surface-modified cellulose dialysis membrane containing alpha-tocopherol	• manufactures *in vitro* testing shows favorable effect profile with respect to blood contact phase activation, interleukin 6 production and reactive oxygen production compared to selected other dialysis membranes	[12]		
				• in hemodialysis patients a meta-analysis that included studies comparing different types of vitamin E-modified dialysis membranes to control dialysis membranes suggested reduced oxidative stress and inflammation status	[13]
reducing gut-derived uremic toxins	*resistant starch*	• prebiotic,<br>• amelioration of oxidative stress in kidney tissue (downregulation of reactive oxygen species-generating proteins, reduction of	[14]	• in hemodialysis patients reduction of gut-derived uremic toxins (significant for free concentrations of indoxyl sulfate in plasma)	[15]

		nitrotyrosine, upregulation of antioxidant proteins),<br>• restoration of gut epithelial tight junctions			
	AST-120	• oral sorbent,<br>• reduction of plasma indoxyl sulfate concentration,<br>• reduction of superoxide production in skeletal muscle	[16]	• in patients with advanced CKD possible positive impact of > 6 month AST-120 treatment on left cardiac ventricle geometry (cross-sectional study)<br><br>• in patients with CKD stage 3/4 no impact of AST-120 long-term treatment on renal disease progression or mortality (on top of RAAS inhibition and intended low protein diet)	[17]<br><br>[18]
exercise training	*long-term aerobic exercise training*	• decrease of renal superoxide production and oxidative protein damage<br>• increased activity of renal antioxidant enzymes	[19]	• in CKD patients with mild to moderately reduced eGFR reduction of serum lipid oxidation products and increase of the reduced form of glutathione<br><br>• in CKD patients with mild to moderately reduced eGFR after 10 years less frequent occurrence of death or initiation of renal replacement therapy (group size n=7 and n=9)	[20]<br><br>[21]
	long-term intradialytic exercise training			• in hemodialysis patients reduction of oxidative stress related parameters (alkaline phosphatase, thiobarbituric acid reactive substances)<br><br>• in hemodialysis patients reduction of lipid peroxidation (15-F(2)alpha-isoprostanes)	[22]<br><br>[23]

CKD chronic kidney disease
CVD cardiovascular disease
RAAS renin-angiotensin-aldosterone system

References

[1] Yates MS, Tauchi M, Katsuoka F, Flanders KC, Liby KT, Honda T, Gribble GW, Johnson DA, Johnson JA, Burton NC, Guilarte TR, Yamamoto M, Sporn MB, Kensler TW. Pharmacodynamic characterization of chemopreventive triterpenoids as exceptionally potent inducers of Nrf2-regulated genes. Mol Cancer Ther. 2007;6:154-62. PMID: 17237276.

[2] Vaziri ND, Liu S, Farzaneh SH, Nazertehrani S, Khazaeli M, Zhao YY. Dose-dependent deleterious and salutary actions of the Nrf2 inducer dh404 in chronic kidney disease. Free Radic Biol Med. 2015;86:374-381. doi: 10.1016/j.freeradbiomed.2015.04.022.

[3] Pergola PE, Raskin P, Toto RD, Meyer CJ, Huff JW, Grossman EB, Krauth M, Ruiz S, Audhya P, Christ-Schmidt H, Wittes J, Warnock DG; BEAM Study Investigators. Bardoxolone methyl and kidney function in CKD with type 2 diabetes. N Engl J Med. 2011;365:327-36. doi: 10.1056/NEJMoa1105351.

[4] de Zeeuw D, Akizawa T, Audhya P, Bakris GL, Chin M, Christ-Schmidt H, Goldsberry A, Houser M, Krauth M, Lambers Heerspink HJ, McMurray JJ, Meyer CJ, Parving HH, Remuzzi G, Toto RD, Vaziri ND, Wanner C, Wittes J, Wrolstad D, Chertow GM; BEACON Trial Investigators. Bardoxolone methyl in type 2 diabetes and stage 4 chronic kidney disease. N Engl J Med. 2013;369:2492-2503. doi: 10.1056/NEJMoa1306033.

[5] Ates B, Abraham L, Ercal N. Antioxidant and free radical scavenging properties of N-acetylcysteine amide (NACA) and comparison with N-acetylcysteine (NAC). Free Radic Res. 2008;42:372-377. doi: 10.1080/10715760801998638.

[6] Whillier S, Raftos JE, Chapman B, Kuchel PW. Role of N-acetylcysteine and cystine in glutathione synthesis in human erythrocytes. Redox Rep. 2009;14:115-124. doi: 10.1179/135100009X392539.

[7] Henze A, Raila J, Scholze A, Zidek W, Tepel M, Schweigert FJ. Does N-acetylcysteine modulate post-translational modifications of transthyretin in hemodialysis patients? Antioxid Redox Signal. 2013;19:1166-1172. doi: 10.1089/ars.2012.5125.

[8] Tepel M, van der Giet M, Statz M, Jankowski J, Zidek W. The antioxidant acetylcysteine reduces cardiovascular events in patients with end-stage renal failure: a randomized, controlled trial. Circ. 2003;107:992-995. PMID: 12600912.

[9] Nascimento MM, Suliman ME, Silva M, Chinaglia T, Marchioro J, Hayashi SY, Riella MC, Lindholm B, Anderstam B. Effect of oral N-acetylcysteine treatment on plasma inflammatory and oxidative stress markers in peritoneal dialysis patients: a placebo-controlled study. Perit Dial Int. 2010;30:336-342. doi:10.3747/pdi.2009.00073.

[10] Serbinova E, Kagan V, Han D, Packer L. Free radical recycling and intramembrane mobility in the antioxidant properties of alpha-tocopherol and alpha-tocotrienol. Free Radic Biol Med. 1991;10:263-275. PMID: 1649783.

[11] Boaz M, Smetana S, Weinstein T, Matas Z, Gafter U, Iaina A, Knecht A, Weissgarten Y, Brunner D, Fainaru M, Green MS. Secondary prevention with antioxidants of cardiovascular disease in endstage renal disease (SPACE): randomised placebo-controlled trial. Lancet. 2000;356:1213-1218.

[12] Sasaki M, Hosoya N, Saruhashi M. Development of vitamin E-modified membrane. Contrib Nephrol. 1999;127:49-70. PMID: 10629776.

[13] Yang SK, Xiao L, Xu B, Xu XX, Liu FY, Sun L. Effects of vitamin E-coated dialyzer on oxidative stress and inflammation status in hemodialysis patients: a systematic review and meta-analysis. Ren Fail. 2014;36:722-731. doi: 10.3109/0886022X.2014.890858.

[14] Vaziri ND, Liu SM, Lau WL, Khazaeli M, Nazertehrani S, Farzaneh SH, Kieffer DA, Adams SH, Martin RJ. High amylose resistant starch diet ameliorates oxidative stress, inflammation, and progression of chronic kidney disease. PLoS One. 2014;9:e114881. doi: 10.1371/journal.pone.0114881. eCollection 2014.

[15] Sirich TL, Plummer NS, Gardner CD, Hostetter TH, Meyer TW. Effect of increasing dietary fiber on plasma levels of colon-derived solutes in hemodialysis patients. Clin J Am Soc Nephrol. 2014;9:1603-1610. doi: 10.2215/CJN.00490114.

[16] Nishikawa M, Ishimori N, Takada S, Saito A, Kadoguchi T, Furihata T, Fukushima A, Matsushima S, Yokota T, Kinugawa S, Tsutsui H. AST-120 ameliorates lowered exercise capacity and mitochondrial biogenesis in the skeletal muscle from mice with chronic kidney disease via reducing oxidative stress. Nephrol Dial Transplant. 2015;30:934-942. doi: 10.1093/ndt/gfv103.

[17] Nakai K, Fujii H, Kono K, Goto S, Fukagawa M, Nishi S. Effects of AST-120 on left ventricular mass in predialysis patients. Am J Nephrol. 2011;33:218-223. doi: 10.1159/000324354.

[18] Cha RH, Kang SW, Park CW, Cha DR, Na KY, Kim SG, Yoon SA, Han SY, Chang JH, Park SK, Lim CS, Kim YS. A randomized, controlled trial of oral intestinal sorbent AST-120 on renal function deterioration in patients with advanced renal dysfunction. Clin J Am Soc Nephrol. 2016;11:559-567. doi: 10.2215/CJN.12011214.

[19] de Souza PS, da Rocha LG, Tromm CB, Scheffer DL, Victor EG, da Silveira PC, de Souza CT, Silva LA, Pinho RA. Therapeutic action of physical exercise on markers of oxidative stress induced by chronic kidney disease. Life Sci. 2012;91:132-136. doi: 10.1016/j.lfs.2012.06.028.

[20] Pechter U, Ots M, Mesikepp S, Zilmer K, Kullissaar T, Vihalemm T, Zilmer M, Maaroos J. Beneficial effects of water-based exercise in patients with chronic kidney disease. Int J Rehabil Res. 2003;26:153-156. PMID: 12799612.
[21] Pechter Ü, Raag M, Ots-Rosenberg M. Regular aquatic exercise for chronic kidney disease patients: a 10-year follow-up study. Int J Rehabil Res. 2014;37:251-255. doi: 10.1097/MRR.0000000000000063.
[22] Wilund KR, Tomayko EJ, Wu PT, Ryong Chung H, Vallurupalli S, Lakshminarayanan B, Fernhall B. Intradialytic exercise training reduces oxidative stress and epicardial fat: a pilot study. Nephrol Dial Transplant. 2010;25:2695-2701. doi: 10.1093/ndt/gfq106.
[23] Groussard C, Rouchon-Isnard M, Coutard C, Romain F, Malardé L, Lemoine-Morel S, Martin B, Pereira B, Boisseau N. Beneficial effects of an intradialytic cycling training program in patients with end-stage kidney disease. Appl Physiol Nutr Metab. 2015;40:550-556. doi: 10.1139/apnm-2014-0357.
